# How to Assess *in vitro* Probiotic Viability and the Correct Use of Neutralizing Agents

**DOI:** 10.3389/fmicb.2020.00204

**Published:** 2020-03-03

**Authors:** Luca Grispoldi, Riccardo Giglietti, Giovanna Traina, Beniamino Cenci-Goga

**Affiliations:** ^1^Department of Veterinary Medicine, University of Perugia, Perugia, Italy; ^2^Department of Pharmaceutical Sciences, University of Perugia, Perugia, Italy; ^3^NoNit srl, University of Perugia, Perugia, Italy

**Keywords:** probiotic, bactericidal activity, gastric juice, neutralization, viability

## Abstract

Probiotic viability is generally determined by quantifying its resistance to simulated gastric juice or to simulated intestinal fluid in *in vitro* tests, which measure microbial survival after given periods of contact. The use of a neutralizing agent is needed to avoid a carry-over of gastric or intestinal juice into the culture media of the subsequent analysis and to avoid any antimicrobial effect extended over the defined period of contact of the test. Neutralization of gastric juice and intestinal juice are of the utmost importance to present data accurately. Failing to do so determines a carry-over of bactericidal activity to the plates used for the enumeration, which further reduces the number of surviving cells. Examples of such incorrect adaptation of the test are available in literature. The purpose of this perspective stems from the discovery that many studies do not adhere to internationally recognized standards, e.g., EN 1040:2005 (European Committee for Standardization [ECS], 2005), to evaluate the basic, bactericidal activity of compounds, especially for the neutralization step.

## Introduction

The term “probiotic” means “for life” and is generally used to identify bacteria that can exert a beneficial effect to humans and animals. The first observations are attributed to the Nobel Prize laureate Élie Metchnikoff who, in 1907, proposed that “*The dependence of the intestinal microbes on the food makes it possible to adopt measures to modify the flora in our bodies and to replace the harmful microbes by useful microbes*” (Metchnikoff, 1908). The definition has more recently been reworded as “*live microorganisms that, when administered in adequate amounts confer a health benefit on the host*” (Food and Agriculture Organization of the United Nations, 2002). 90 years after Metchnikoff, the term “prebiotic” was first defined as “*a non-digestible food ingredient that beneficially affects the host by selectively stimulating the growth and/or activity of one or a limited number of bacteria in the colon, and thus improves host health*” (Gibson and Roberfroid, 1995). Today there are at least three criteria to classify a food ingredient as prebiotic: (i) resistance to gastric acidity, to hydrolysis by mammalian enzymes, and to gastrointestinal absorption; (ii) fermentation by intestinal microflora; and (iii) selective stimulation of the growth and/or activity of those intestinal bacteria that contribute to health and well-being. There are currently only two food ingredients that fulfill these criteria, i.e., inulin and *trans*-galactooligosaccharides (TOS) (Roberfroid, 2007). Two more terms are useful to understand this opinion: “microbiota,” which is a collective term for the microorganisms that live in or on the human body (specific clusters of microbiota are found on the skin or in the gastrointestinal tract, mouth, vagina, and eyes) and “microbiome,” which comprises all the genetic material within a microbiota (the entire collection of microorganisms in a specific niche, such as the human gut) and which can also be referred to as the metagenome of the microbiota (Rothschild et al., 2018). After the first attempts made by FAO and WHO (Food and Agriculture Organization of the United Nations, 2002, 2006) to provide health and nutritional information and guidelines to evaluate probiotics, in view of the growing popularity of probiotic foods and the lack of international consensus on the methodology to assess their efficacy and safety, several societies and associations have more recently released a number of guidelines or position papers on this topic (Hill et al., 2014; Kolacek et al., 2017). Despite many attempts made by regulatory bodies (Kolacek et al., 2017), the status of probiotic products has not been established on an international basis–there is no label control and there are no periodic screenings of the products’ quality and safety. Apart from the issues related to hygiene and safety and to the taxonomy, nomenclature, and classification of strains (Aureli et al., 2010; Lefevre et al., 2015; Llewellyn and Foey, 2017; Suez et al., 2018; Zmora et al., 2018), research mainly focuses on the viability and survival of commercial probiotic formulations during their passage through the gastro-intestinal (GI) tract (Drago et al., 2004; Cook et al., 2011; Dominici et al., 2011; Sahadeva et al., 2011; Jensen et al., 2012; Fredua-Agyeman and Gaisford, 2014; Vecchione et al., 2018). Whereas scientists are unanimous in giving the basic properties for oral probiotics as the ability to (i) survive in the acidic environment during gastric transit and (ii) be active and vital in the intestine, there is no consensus on the ability of exogenous bacteria to colonize the human GI tract and, in particular, the mucosa-associated surfaces. Some studies suggest that probiotics are generally shed in stool only for the time of administration and shortly subsequently, whereas others suggest generalized, or subset-specific, probiotic shedding in stool, also after interruption of the administration. Although *in vitro* studies indicate that probiotics can adhere to human intestinal epithelium, these studies are susceptible to bias with regard to bacterial concentrations, the growth stage, incubation time, and culture media used and are not physiological (Suez et al., 2018; Zmora et al., 2018). However, whatever the consensus is on colonization, acid and bile resistance are characteristics used to select potential probiotic strains. In fact, although probiotic formulations have to contain a sufficient number of living microorganisms (Kolacek et al., 2017), it is of the utmost importance for the release of bacteria from the formulation to be controlled and their deposition to be spread along the intestine in order to deliver probiotics to both small and large intestine. To this end, many *in vitro* studies have been conducted, and the results show heterogeneous behavior, depending on the species or strains analyzed and methods applied (Chandramouli et al., 2004; Lin et al., 2006; Cook et al., 2011; Sahadeva et al., 2011; Jensen et al., 2012; Fredua-Agyeman and Gaisford, 2014; Li et al., 2019).

The purpose of this perspective stems from the discovery that many studies do not adhere to internationally recognized standards, e.g., EN 1040:2005 (European Committee for Standardization [ECS], 2005), to evaluate the basic, bactericidal activity of compounds, especially for the neutralization step.

## Assessment of Probiotic Viability *In vitro*

Probiotic viability is generally determined by quantifying its resistance to simulated gastric juice or to simulated intestinal fluid in *in vitro* tests, which measure microbial survival after given periods of contact, i.e., [log colony forming units (cfu) of surviving viable cells]/[log cfu of initial viable cells inoculated]. The test is basically performed by (i) inoculating aliquots of probiotic suspensions (at known log cfu viable cells) into the simulated juices, (ii) incubating for the given periods of contact, (iii) serial or decimal diluting of aliquots of the suspension of juice and probiotic, (iv) plating into appropriate culture media and incubating at the given incubation conditions, and (v) calculating the number of residual cfu. Unfortunately, this is not sufficient. Without neutralizing the juice correctly, step (iii) above has the effect of a carry-over of juice into the culture media, with the antimicrobial effect extended well over the defined period of contact of the test. In fact, the effect of any bactericidal activity of the simulated gastric and intestinal juices endures throughout the entire plate incubation period (step iv above).

### Assay System

Any assay that measures the resistance of probiotics to simulated gastric or intestinal juice is analogous to the evaluation of bactericidal activity of any given disinfectant or antiseptic against bacterial reference tester strains. In fact, the objective of the test is to determine the number of residual bacteria after a specified time of contact between a suspension with bactericidal activity and the microorganism. In our case, the tester strains are the probiotic formulations and the bactericidal suspensions are the gastric or intestinal juice. For this purpose, therefore, the experimental procedure requires the following elements: (a) microorganism suspension at known concentration (the probiotic strain or formulation), (b) bactericidal suspension or solution (the simulated gastric or intestinal juice), and (c) neutralizer with buffer and adsorbing capacity (a neutralizer is usually used for the isolation of microorganisms from sanitized environmental surfaces in a laboratory setting, and in the case of gastric and intestinal juice, it is also required to hold buffering capacity to bring the pH of the juice to neutral).

### Execution of the Assay

Probiotic suspension, simulated gastric, and intestinal juice preparation and neutralizer formulation, together with test temperature and contact time, are arranged according to the specific requirements of the test (see section “Original Data – Assay”). In this case, we want to draw the readers’ attention to the step required for a correct *in vitro* assessment of probiotic viability after ingestion. The evaluation of methods based on a physical barrier against adverse environmental conditions, e.g., microencapsulation or the use of gastro resistant capsules (Cook et al., 2011), is beyond the scope of this perspective.

#### Preliminary Assay

A preliminary assay is required to (i) calculate the count of the probiotic suspension (results are expressed in cfu ml^–1^ and the “Nv” value is calculated); (ii) validate the non-toxicity of the liquid media used for serial or decimal dilutions of probiotic suspension (before serial or decimal dilutions, the bacterial suspension is left in contact with the diluent at the temperature adopted during the essay for the longest period to be tested, the results are expressed in cfu ml^–1^, and the “A” value is calculated); (iii) validate neutralizer non-toxicity (the bacterial suspension is left in contact with the neutralizer at the temperature adopted during the essay for the longest period to be tested, the results are expressed in cfu ml^–1^, and the “B” value is calculated); and (iv) validate gastric and intestinal juice non-toxicity after neutralization (gastric or intestinal juice is put in contact with the neutralizer at the temperature adopted during the essay and for the period required for neutralization). The bacterial suspension is then left in contact with the “neutralized gastric or intestinal juice” at the temperature adopted during the essay for the longest period to be tested, the results are expressed in cfu ml^–1^ mL, and the “C” value is calculated.

#### Assay

On the day of the assay, the bacterial suspension is analyzed, the results are expressed in cfu ml^–1^, and the “N” value is calculated. The gastric or intestinal fluid, bacterial suspension, diluent, and neutralizer are stabilized at the given test temperature (usually at 37°C). One test tube containing the diluent and the bacterial suspension is prepared for each probiotic and for each artificial fluid to be tested. It is stabilized at the test temperature and then added with the fluid to be tested for the selected times at the test temperature. At the end of each contact time, aliquots of the mixture are transferred into a test tube containing the neutralizer. After the time required for neutralization, the mixture is analyzed, the results are expressed in cfu ml^–1^, and the “Na” value is calculated.

#### Calculation and Expression of Results

The calculation of the bacterial count for the suspension test (N, bacterial counting for the probiotic before contact with the juice), for the bacterial count for the assay (Na, bacterial counting for the probiotic at the end of the contact time), and for the preliminary assay (A, B, C, and Nv) is performed by applying standard microbiological methods. The calculation of vitality reduction is expressed in a logarithm and is calculated for each organism and test concentration using the formula:

log⁡R=log⁡N-log⁡N⁢a.

#### Assay Validity Criteria

No statistically significant differences between N, Nv, A, B, and C should be detected. On this point, the European Standard EN 1040 (European Committee for Standardization [ECS], 2005) establishes that the differences between N, Nv, A, B, and C have to be within the range of ± 0.05 times for data expressed as cfu ml^–1^, regardless of any statistical evaluation. As an example, Table 1 shows the result from such tests routinely carried out at our laboratory: Counts for probiotic challenged with diluent, neutralizer, and neutralized gastric juice are within the range of ± 0.05 times for data expressed as cfu ml^–1^.

**TABLE 1:** Assay validity for simulated gastric juices A and B. Differences between counts must be within the range of ± 0.05 times for data expressed as cfu/ml^–1^.

	mean (cfu/ml^–1^)	*Sd*	Nv	N	A	B	Ca	Cb
*Saccharomyces cerevisiae*								
			7.92	8.28	7.97	7.91	8.16	7.93
Nv	7.92	0.46	0.00	−0.05	−0.01	0.00	−0.03	0.01
N	8.28	0.74	0.04	0.00	0.04	0.04	0.01	0.05
A	7.97	0.68	0.01	−0.04	0.00	0.01	−0.02	0.02
B	7.91	0.72	0.00	−0.05	−0.01	0.00	−0.03	0.01
Ca	8.16	0.78	0.03	−0.01	0.02	0.03	0.00	0.04
Cb	7.93	0.26	0.00	−0.04	−0.01	0.00	−0.03	0.01
*Lactobacillus acidophilus*								
			7.30	7.10	7.20	7.05	7.21	7.43
Nv	7.30	0.39	0.00	0.03	0.01	0.03	0.01	−0.02
N	7.10	0.50	−0.03	0.00	−0.01	0.01	−0.02	−0.05
A	7.20	0.57	−0.01	0.01	0.00	0.02	0.00	−0.03
B	7.05	0.17	−0.04	−0.01	−0.02	0.00	−0.02	−0.05
Ca	7.21	0.52	−0.01	0.02	0.00	0.02	0.00	−0.03
Cb	7.43	0.35	0.02	0.04	0.03	0.05	0.03	0.00
*Enterococcus faecium*								
			8.39	8.62	8.63	8.43	8.78	8.69
Nv	8.39	0.44	0.00	−0.04	−0.01	0.00	−0.03	−0.04
N	8.62	0.40	0.04	0.00	0.04	0.04	0.01	−0.01
A	8.63	0.48	0.01	−0.04	0.00	0.01	−0.02	−0.01
B	8.43	0.43	0.00	−0.04	−0.01	0.00	−0.03	−0.03
Ca	8.78	0.55	0.03	−0.01	0.02	0.03	0.00	0.01
Cb	8.69	0.50	0.03	0.01	0.01	0.03	−0.01	0.00

*Nv: count of the probiotic suspension; N: count of the probiotic suspension on the day of the test; A: validation of the non-toxicity of the liquid media used for serial or decimal dilutions of probiotic suspension, i.e., counts of the probiotic suspension after being left in contact with the diluent at the temperature adopted during the essay for the longest period to be tested; B: validation of the neutralizer non-toxicity, i.e., counts of the bacterial suspension after being left in contact with the neutralizer at the temperature adopted during the essay for the longest period to be tested; C: validation of the gastric juice non-toxicity after neutralization, i.e., counts of the bacterial suspension after being left in contact with gastric juice neutralized at the temperature adopted during the essay and for the period required for neutralization (Ca: simulated gastric juice A; Cb: simulated gastric juice B).*

#### Original Data – Assay

The method described above has been successfully applied to test the viability of a commercial probiotic against two formulations of gastric juice. The first (“gastric juice A”) was a solution of 0.07 N hydrochloric acid with pH 1.5 at 37°C, as specified by the American Society of Testing Materials (American Society of Testing Materials [ASTM], 2003). The second (“gastric juice B”) consisted of 0.03 M sodium chloride, 0.084 M hydrochloric acid, and 0.32% (w/v) pepsin with pH 1.4 at 37°C, as recommended by the U.S. Pharmacopeia (U.S. Pharmacopeia and National Formulary, 2003).

Each strain of the commercial formulation Enterelle (Bromatech srl, Milano, Italy) (*Saccharomyces cerevisiae* var boulardii MTCC-5375, *Enterococcus faecium* UBEF-41, and *Lactobacillus acidophilus* LA 14) were grown aerobically in Nutrient Broth (NB; CM0001, Oxoid, Basingstoke, United Kingdom) at 37°C for 24 h. The total viable cell count on Nutrient Agar (NA; CM0003, incubated at 37°C on air for 24 h; Oxoid) at 24 h was approximately 10^9^ cfu ml^–1^. Total viable cell counts for all dilutions were recorded as controls on NA and on the following media. MRS Agar (Oxoid), pH 5.5, at 30°C for 72 h under anaerobic conditions (Gas generating kit, Oxoid), was used for counts of *L. acidophilus* LA 14; Enterococcus agar (CM0984, Oxoid) at 37°C for 48 h was used for *E. faecalis*; and DRBC (Dichloran Rose Bengal Chloramphenicol, 4013932 Biolife, Milan, Italy) at 25°C for up to 7 days was used for *S. cerevisiae* var boulardii.

Before each test, each strain was checked for purity and confirmed: (i) *Enterococcus faecium* UBEF-41 by cell morphology after Gram staining (Gram positive cocci), presence of catalase, growth on bile-aesculin-azide agar (Coccosel agar, BioMéhrieux), non-hemolytic on tryptic soy agar (Biolife), to which 5% of ram blood had been added; (ii) *Lactobacillus acidophilus* LA 14, by cell morphology after Gram staining (Gram positive bacilli), presence of catalase; (iii) *Saccharomyces cerevisiae* var boulardii by cell morphology after Gram staining (unicellular, globose, and ellipsoid to elongate in shape with a diameter of 2–8 μm and length of 3–25 μm, pseudohyphae, if present, are rudimentary, hyphae are absent).

Decimal dilutions were performed to obtain the following concentrations in NB: 10^9^, 10^8^, 10^7^, 10^6^, 10^5^, 10^4^, 10^3^, and 10^2^ cfu ml^–1^. Counting in agar in triplicate was performed. The number of cfu per ml of the suspension was determined following incubation (see above for the incubation conditions), and *N* value was calculated.

Vitality reduction activity was performed according to the BS EN 1040:2005 (European Committee for Standardization [ECS], 2005) using a specific neutralizer to halt the antibacterial activity of “gastric juice A” (ASTM) and “gastric juice B” (U.S. Pharmacopeia) at any given time. The neutralizer of choice (patent pending) was a modification of the original formulation of Engley and Dey (1970) with the addition of a pepsin adsorbing agent and pH-buffering capacity. The assay was validated to determine the experimental conditions, the neutralizer non-toxicity, and the dilution-neutralization test according to paragraphs 2.2.1 to 2.2.4 above. The assay sample and the bacterial suspensions had previously been stabilized at the test temperature of 37°C ± 1°C. For each bacterial strain and for each concentration of the test substance, one test tube containing 1 ml of sterile saline and 1 ml of bacterial test suspension was prepared at the temperature adopted during the assay. After 2 min of contact, 8 ml of the sample extracts “gastric juice A” (ASTM) and “gastric juice B” (U.S. Pharmacopeia), respectively, were added and left in contact for the selected times at the test temperature. At the end of the contact time, 1 ml of mixture was transferred into a test tube containing 8 ml of neutralizer and 1 ml of sterile saline. After 5 min of neutralization procedure, the mixture was vortex-stirred and count in triplicate was performed.

The number of cfu per plate was determined following incubation (see above for the incubation conditions) and Na value was then calculated. Prism, version 6.0 h, for Mac OS X (GraphPad, San Diego, CA, United States) was used for the graphs and to analyze the data by one-way ANOVA. A *P-*value of <0.05 was considered to be significant. Counts were performed using the number of colonies counted on triplicate. Only the plates showing a number of colonies included in a 15–300 range were used to perform the result calculation. A deviation of 10% is accepted, so the limits are 14 and 330. In the assay, where the number of cfu on every plate counted is <14, the number of cfu ml^–1^ was recorded as <1.4 × 10^2^. Where the number of cfu on every plate counted was >330, the number of cfu ml^–1^ was recorded as >3.3 × 10^3^ (Rossitto et al., 2012).

#### Original Data – Results and Discussion

The assay was correctly validated for both “gastric juice A” and “gastric juice B” (Table 1) because the differences between N, Nv, A, B, and C were within the range of ± 0.05 times for data expressed as cfu ml^–1^. Moreover, no statistically significant differences were observed between the groups by ordinary one-way ANOVA. The vitality reduction is shown in Table 2. Briefly, vitality reduction for *S. cerevisiae* after contact with simulated “gastric juice A” ranged from 1.27 to 2.03 log cfu ml^–1^; for *L. acidophilus*, from 0.46 to 7.1; and for *E. faecium*, from 2.72 to 3.77 (Table 2). The vitality reduction for *S. cerevisiae* after contact with simulated «gastric juice B» ranged from 1.40 to 3.29 log cfu ml^–1^; for *L*. *acidophilus*, from -0.28 to 7.1; and for *E*. *faecium*, from 5.94 to 6.90 (Table 2).

**TABLE 2:** Vitality reduction (R) after contact with simulated “gastric juice A” (A) and “gastric juice B” (B).

	mean (cfu/ml^–1^)	*sd*	vitality reduction (R)
**(A)**			

*Saccharomyces cerevisiae*			
N	8.28	0.74	
Na 30’	6.40^a^	0.99	1.88
Na 60’	6.43^a^	0.32	1.85
Na 90’	6.25^a^	0.36	2.03
Na 120’	7.01^a^	1.21	1.27
*Lactobacillus acidophilus*			
N	7.10^a^	0.50	
Na 30’	6.64^a^	0.52	0.46
Na 60’	2.67	0.39	4.43
Na 90’	1.57	0.39	5.53
Na 120’	0.00	0.00	7.10
*Enterococcus faecium*			
N	8.62	0.40	
Na 30’	5.90^ab^	0.58	2.72
Na 60’	5.86^a^	1.08	2.76
Na 90’	5.75^ab^	0.59	2.87
Na 120’	4.85^b^	0.51	3.77

**(B)**			

*Saccharomyces cerevisiae*			
N	8.28	0.74	
Na 30’	6.88^a^	0.37	1.40
Na 60’	6.15^ab^	0.51	2.13
Na 90’	5.51^ab^	0.11	2.78
Na 120’	4.99^b^	0.27	3.29
*Lactobacillus acidophilus*			
N	7.10	0.50	
Na 30’	7.38	0.45	−0.28
Na 60’	7.41	0.22	−0.31
Na 90’	6.84	0.25	0.26
Na 120’	0.00^a^	0.00	7.10
*Enterococcus faecium*			
N	8.62	0.40	
Na 30’	2.60^ab^	0.50	6.02
Na 60’	2.68^a^	0.43	5.94
Na 90’	1.80^bc^	0.42	6.82
Na 120’	1.73^c^	0.55	6.90

*Values calculated as log*R* = log*N* – log*Na*. **N:** counts of the probiotic suspension on the day of the test; **Na:** counts after the given period of contact with gastric juice (30, 60, 90, and 120 min). ^*abc*^Different superscripts in the same column and for each microorganism indicate significant different means (*p* < 0.05).*

*Saccharomyces cerevisiae* showed a very good resistance to both simulated gastric juices used in the test; even after 120 min of contact, *L. acidophilus* was resistant to gastric juice B up to 90 min and then was no longer detectable at 120 min, but showed a lesser resistance to gastric juice A, with a vitality reduction of more than 4 log cfu ml^–1^ after 60 min of contact. On the other hand, *E. faecium* was more sensitive to gastric juice B. Our results are quite different from the data obtained by Vecchione et al. (2018) that described significant reductions for the same commercial product already after 30 min of incubation, probably as a consequence of the lack of neutralization. For both studies, however, the simulated gastric juice prepared according to the indications of the American Society of Testing Materials (gastric juice A) produced a higher vitality reduction than the one prepared according to the U.S. Pharmacopeia (gastric juice B).

## Comments

Neutralization of gastric juice and intestinal juice is of the utmost importance to present data accurately. Failing to do so determines a carry-over of bactericidal activity to the plates used for the enumeration, which further reduces the number of surviving cells. Examples of such incorrect adaptations of the test are available in literature (Chandramouli et al., 2004; Lin et al., 2006; Sahadeva et al., 2011; Jensen et al., 2012; Fredua-Agyeman and Gaisford, 2014; Vecchione et al., 2018). These results are therefore biased, as they do not represent the effect of gastric or intestinal juice for the given amount of time. Instead, they show the effect for the entire duration of plate incubation after the specified and studied contact time between the probiotic and the juice. To neutralize the formulations used in this work, a modification of the original formulation of Engley and Dey (1970) with the addition of a pepsin-adsorbing agent was developed. Examples of pepsin-adsorbing agents are aluminum hydroxide gel and charcoal (Piper and Fenton, 1961).

Some authors argue that decimal solution in sterile saline solution exponentially reduce the impact of the carry-over (Lin et al., 2006; Sahadeva et al., 2011); however, carry-over from the solution to the agar plate is particularly important when microorganisms are submitted to the harsher conditions. In particular, when lower or no-dilution are performed, the effect of the stressor could determine an important underestimation of viable cells. Other authors add centrifugations and washing steps to reduce the carry-over (Lin et al., 2006; Sahadeva et al., 2011; Russo et al., 2012), but the number of steps required (at least three) and the related maneuvers increase the risk of contamination and do not guarantee the complete absence of carry-over. Moreover, the European Committee for Standardization (European Committee for Standardization [ECS], 2005) advises against this procedure. Whatever the procedure of choice, an assay validity test, such as that explained in Sections “Preliminary Assay, Assay, Calculation and Expression of Results, and Assay Validity Criteria,” is necessary. Moreover, when testing for resistance of probiotics to simulated gastric or intestinal juices, it is of the utmost importance to take into account not only the buffering capacity of the neutralizing agent of choice, but also the ability to contrast the effect of typical stress conditions of the oro-gastrointestinal tract (i.e., lysozyme, pepsine, pancreatine, and bile salts).

## Data Availability Statement

The raw data supporting the conclusions of this article will be made available by the authors, without undue reservation, to any qualified researcher.

## Author Contributions

BC-G, GT, and LG contributed to the conception and design of the study. BC-G wrote the first draft and finalized the manuscript. RG wrote sections of the manuscript. All authors contributed to the manuscript revision and read and approved the submitted version.

## Conflict of Interest

BC-G is CEO of the company NoNit srl. The remaining authors declare that the research was conducted in the absence of any commercial or financial relationships that could be construed as a potential conflict of interest.
